# Etymologia: *Mycobacterium abscessus* subsp. *bolletii*

**DOI:** 10.3201/eid2003.ET2003

**Published:** 2014-03

**Keywords:** etymologia, Mycobacterium abscessus, Mycobacterium abscessus subsp. bolletii, massiliense, bacteria, mycobacteria, abscess, Claude Bollet, Marseille

## *Mycobacterium abscessus* subsp. *bolletii*

From the Latin *ab-* (“away”) + *cedere* (“to go”), an abscess is named for the notion that humors leave the body through pus. *Mycobacterium abscessus* was first isolated from gluteal abscesses in a 62-year-old patient who had injured her knee as a child and had a disseminated infection 48 years later. The species *M. bolletii*, named after the late microbiologist and taxonomist Claude Bollet, was described in 2006. In current taxonomy, *M. bolletii* and *M.* *massiliense* (named for Massilia, the ancient Greek and Roman name for Marseille, where the organism was isolated) have been incorporated into *M. abscessus* subsp. *bolletii*. 

## References

[1] Adékambi T, Berger P, Raoult D, Drancourt M. *rpoB* gene sequence-based characterization of emerging non-tuberculous mycobacteria with descriptions of *Mycobacterium bolletii* sp. nov., *Mycobacterium phocaicum* sp. nov. and *Mycobacterium aubagnense* sp. nov. Int J Syst Evol Microbiol. 2006;56:133–43 . 10.1099/ijs.0.63969-0 16403878

[2] Adékambi T, Reynaud-Gaubert M, Greub G, Gevaudan MJ, La Scola B, Raoult D, Amoebal coculture of “*Mycobacterium massiliense*” sp. nov. from the sputum of a patient with hemotoic pneumonia. J Clin Microbiol. 2004;42:5493–501 . 10.1128/JCM.42.12.5493-5501.2004 15583272 PMC535245

[3] Leao SC, Tortoli E, Euzéby JP, Garcia MJ. Proposal that *Mycobacterium massiliense* and *Mycobacterium bolletii* be united and reclassified as *Mycobacterium abscessus* subsp. *bolletii* comb. nov., designation of *Mycobacterium abscessus* subsp. *abscessus* subsp. nov. and emended description of *Mycobacterium abscessus.* Int J Syst Evol Microbiol. 2011;61:2311–3 and. 10.1099/ijs.0.023770-0 21037035

[4] Moore M, Frerichs JB. An unusual acid-fast infection of the knee with subcutaneous, abscess-like lesions of the gluteal region. J Invest Dermatol. 1953;20:133–69 . 13035193 10.1038/jid.1953.18

